# The effects of bilingualism on cognition and behaviour in individuals with attention deficits: A scoping review

**DOI:** 10.3389/fpsyg.2022.1057501

**Published:** 2022-12-23

**Authors:** Franziska Köder, Curtis Sharma, Sarah Cameron, Maria Garraffa

**Affiliations:** ^1^Center for Multilingualism in Society Across the Lifespan, Department of Linguistics and Scandinavian Studies, University of Oslo, Oslo, Norway; ^2^University of Cambridge, Cambridge, United Kingdom; ^3^Faculty of Medicine and Health Sciences, East Anglia University, Norwich, United Kingdom

**Keywords:** bilingualism, multilingualism, ADHD, attention, executive function

## Abstract

**Background:**

Weaknesses in executive function have persistently been found to be associated with Attention Deficit Hyperactivity Disorder (ADHD), while bilinguals have been argued to show advantages in executive functions. While there has been some research into how bilingualism affects cognitive skills and behaviour in individuals with attention deficits, the question is still very much open. The aim of this systematic review is to gather, synthesise and evaluate existing evidence on how bilingual language experience and attention deficits affect executive function performance and ADHD-related symptoms in children and adults.

**Methods:**

Following PRISMA guidelines, a comprehensive literature search in relevant databases (PsycInfo, PubMed, Scopus, ERIC, Web of Science, EMBASE, MEDLINE, LLBA) was performed using search strings related to attention difficulties/ADHD and bilingualism. All quantitative studies were included that presented original empirical data on the combined effects of bilingualism and attention levels, regardless of age group and methodology. The screening procedure revealed nine relevant studies.

**Results:**

Across the nine identified studies, a total of 2071 participants were tested. Of these, seven studies involved children and two adults. The studies varied considerably with respect to their design and methodology, the targeted executive function skills or behavioural symptoms, as well as their measure of bilingualism and attention levels. Most studies assessed aspects of executive function performance such as interference control, response inhibition, working memory or cognitive flexibility. Three studies looked at the effects of bilingualism on ADHD-related symptoms or ADHD diagnosis. Across the studies, no systematic advantage or disadvantage of bilingualism on cognitive performance or behaviour in people with attention deficits was observed.

**Conclusion:**

The limited number of identified studies provide no consistent evidence that bilingualism alleviates or intensifies attention difficulties in adults or children with ADHD. Based on the current state of research, individuals with ADHD and their families should not be concerned that learning additional languages has a negative impact on functioning or cognitive performance.

**Systematic review registration:**

https://doi.org/10.17605/OSF.IO/PK768.

## 1. Introduction

It is estimated that more people in the world are bilingual than monolingual (Grosjean, 2010). At the same time studies have shown that people from minority backgrounds, who are often speakers of several languages, lag behind those from non-minority backgrounds when it comes to prevalence and treatment of Attention Deficit Hyperactivity Disorder (ADHD) (e.g., Slobodin and Masalha, 2020). Given that ADHD is one of the most common neurodevelopmental disorders in childhood and also present in the adult population (Polanczyk et al., 2007; Faraone et al., 2021), understanding this disparity should be an urgent pursuit. There may be several factors contributing to this imbalance. This includes proficiency in the majority language by reporters or caregivers, cultural expectations of development, or knowledge about ADHD (Stevens et al., 2004; Rothe, 2005; Eiraldi et al., 2006). Another reason could be that people from minority backgrounds often have a migration background and are therefore more likely to grow up with multiple languages. As suggested in the field of bilingualism research (see Antoniou, 2019), being bilingual could potentially improve cognitive abilities related to attention. Looking at the small but scientifically interesting group of bilingual speakers with attention deficits could provide new insights into the existence and extent of a so-called bilingual advantage.

In their systematic review on multilingualism and neurodevelopmental disorders, Uljarević et al. (2016) did not find any studies on the effect of multilingualism in people diagnosed with ADHD. However, since then several studies on the topic have appeared. The aim of this systematic review is to gather and synthesise existing evidence on the effects of bilingualism on the cognitive abilities and ADHD-related symptoms of people with attention deficits. The findings of this review could also be informative for practitioners and multilingual families who might be worried that exposing a child with ADHD to multiple languages might be detrimental to their cognitive development and functioning.

As background for the studies, we first briefly introduce the debate of advantages of bilingualism, discuss the potential association between ADHD and executive function deficits, and outline three possibilities of how bilingual language experience might affect cognition and behaviour in people with attention difficulties.

### 1.1. Bilingualism and advantages in executive function

In this review, we use the term “bilingual” to refer to anyone whose language experience includes two or more languages, and also to include multilinguals throughout the review, unless a specific need to distinguish between bilinguals and other multilinguals arises. We use the term “bilingualism” in a wide sense to cover both the early acquisition of two or more languages as well as second languages acquired later in life, but actively used outside of the classroom.

There is now a large body of literature claiming to have found advantages in cognitive skills for bilinguals, namely a group of skills under the umbrella term *executive function* (EF). The three principal executive function skills investigated in the literature are *inhibition*—the ability to inhibit prepotent responses (*response inhibition*) or task-irrelevant information (*interference control*); *cognitive flexibility—*the ability to switch attention between cognitive tasks; and *working memory* – the ability to store, monitor, manipulate, and update information relevant to an initiated or ongoing cognitive task (cf. Miyake et al., 2000). Evidence for an advantage in inhibition has been found for both children (e.g., Bialystok and Martin, 2004; Martin-Rhee and Bialystok, 2008; Poarch and van Hell, 2012) and adults (e.g., Bialystok et al., 2005; Salvatierra and Rosselli, 2011). Similarly, advantages in cognitive flexibility have been reported for children (e.g., Bialystok and Martin, 2004; Carlson and Meltzoff, 2008) and adults (e.g., Bialystok et al., 2004; Marzecová et al., 2013). Finally, there is some evidence that bilingual language experience might improve working memory capacity in children (Morales et al., 2013), but most studies report little or no effect for children and adults (e.g., Namazi and Thordardottir, 2010; Ratiu and Azuma, 2015; Yang, 2017). These cognitive advantages for bilinguals are said to emerge due to the bilingual's need to constantly monitor and manage both of their languages, as the languages not currently in use cannot be “switched off” (e.g., Spivey and Marian, 1999; Colomé, 2001; Starreveld et al., 2014; Bobb et al., 2020).

The so-called “bilingual advantage hypothesis” is controversial and hotly debated. Several studies have failed to replicate bilingual advantages in inhibition, cognitive flexibility, and working memory for both children and adults (e.g., Paap and Greenberg, 2013; Gathercole et al., 2014; Dick et al., 2019; Timmermeister et al., 2020). Furthermore, several meta-analyses report either no evidence of a bilingual advantage or small effect sizes that disappear when correcting for publication bias (de Bruin et al., 2015; Lehtonen et al., 2018; Paap, 2019; Lowe et al., 2021).

On the other hand, Grundy (2020) and others argue that while there are several reports of null findings, the studies that do find group differences far more often report bilinguals outperforming monolinguals than the other way around, even when controlling for factors related to publication bias and task differences. This could indicate that bilingualism might have small positive effects on cognitive performance, however only for certain groups of bilinguals, under certain conditions, and for certain tasks (Grundy, 2020; Ware et al., 2020). Recent studies point to the importance of making finer-grained distinctions assessing for instance bilinguals' language proficiency and usage, and their differential effects on performance in different executive function tasks (Poarch and Krott, 2019; Grundy, 2020). A higher level of bilingual proficiency seems to be significantly associated with better executive function performance (Pot et al., 2018; Thomas-Sunesson et al., 2018). Furthermore, the usage of different languages in different social contexts (Pot et al., 2018) and switching between two languages in the same environment might also lead to certain cognitive benefits (Hartanto and Yang, 2016). This systematic review aims to add to a more nuanced investigation of a “bilingual advantage” by focusing on bilingual speakers from neurodiverse backgrounds, namely people with attention deficits.

### 1.2. Executive function deficits in ADHD

Attention Deficit Hyperactivity Disorder (ADHD) is one of the most common neurodevelopmental disorders in childhood with estimated prevalence of around 5.29–7.1% of the 18 and under population (Willcutt, 2012; Polanczyk et al., 2014). Difficulties can continue into adulthood, where prevalence is estimated at 2.5% of the adult population (Roberts et al., 2015). ADHD is a clinical umbrella term for a set of behaviours, namely inattentiveness, hyperactivity, and impulsivity, which may or may not occur together (American Psychiatric Association, 2013).

Among several underlying cognitive impairments (cf. Sjöwall et al., 2013), ADHD is commonly linked to weaknesses in key executive function domains such as working memory and inhibitory control (Willcutt et al., 2005; Coghill et al., 2018). However, even though children with ADHD tend to perform below their peers in various executive function measures on a group level, more than 50% of individual children with ADHD do not exhibit executive function deficits (Nigg et al., 2005). Furthermore, effect sizes for group differences in performance on executive function were much smaller (0.46–0.69) than group differences on ADHD symptoms (2.5–4.0) (Willcutt et al., 2005). This refutes strong claims that executive function deficits are the primary cause of ADHD. Rather they seem to be an important component of the complex neuropsychology of ADHD, with potentially multiple pathways leading to similar behavioural symptoms (Sonuga-Barke, 2005).

### 1.3. Interactions between bilingualism and attention deficits

Considering that bilingualism and ADHD have potentially opposing effects on cognition, with bilingualism benefiting while ADHD hindering executive function performance, the question arises how these two factors might interact across the lifespan. In the following, we will sketch three possibilities on how bilingualism might affect executive functions and ADHD-related symptoms in children and adults with attention deficits.

First, it is possible that bilingual speakers with attention problems might experience a bilingual advantage, showing a better ability in executive functions and other cognitive domains, and exhibiting less severe symptoms linked to ADHD than their monolingual peers. This is based on the idea that bilinguals' constant need to selectively attend to one language (potentially suppressing their other language) trains executive function skills (cf. Bialystok, 2015). In other words, being a bilingual might improve overall executive function and offset (some) ADHD-related symptoms.

Opposed to that, being bilingual could be an additional burden for individuals with attention deficits, negatively affecting both executive function performance and inattention symptoms. This could be due to bilinguals needing to allocate parts of their already limited cognitive resources on inhibiting interference from their other language, making them slower and more error-prone in cognitive tasks. If this were the case, we would expect bilinguals with ADHD to show lower executive function abilities and more ADHD-related symptoms compared to their monolingual peers with ADHD.

Given that several studies have failed to replicate findings of a bilingual advantage (as noted in Section Bilingualism and advantages in executive function), as well as the small or null effects reported in several meta-analyses (Lehtonen et al., 2018; Paap, 2019), the hypothesis that bilinguals and monolinguals with attention deficits do not differ in cognitive or behavioural aspects is also a strong competitor. In this case, we would expect to find an association between ADHD and executive function deficits, but no effect or interaction with bilingual language proficiency or use.

## 2. Methods

To locate relevant studies on the joint effects of bilingualism and attention deficits, we performed a comprehensive search in June 2021 in databases connected to psychological, clinical, and linguistic research. These include PsycInfo, PubMed, Scopus, ERIC, Web of Science, EMBASE, MEDLINE, and Linguistics and Language Behaviour Abstracts (LLBA). As we expected relatively few relevant hits, the search was done with no restrictions regarding publication year or language. The search string consisted of several keywords relevant to attention difficulties and attentional abilities, and to bilingualism and multilingualism. Three different strings were tested and further expanded with new terms, resulting in the following search string:

[(ADHD or “attention deficit” or “Attention-Deficit” or “attention problem^*^” or “attention difficult^*^” or “attentional abilit^*^” or “attention abilit^*^”) and (bilingual^*^ or multilingual^*^ or “dual language” or “second language” or “minority language” or “home language” or “heritage language”)].

After removing duplicates, the remaining 779 titles were screened based on title and abstract. Screening was done independently by FK and SC, using the online systematic review tool Rayyan (Ouzzani et al., 2016) for efficient collaboration. In case of disagreement or uncertainty (title classified as “maybe” by one or both reviewers), a joint decision was reached during discussion. Given the scarcity of research on the combination of attention deficits and bilingualism, we included all empirical studies presenting original data on a combined effect of the two, regardless of age group and methodology. Exclusion criteria can be summarised in two main categories: (1) off topic, which includes any paper not directly dealing with the combination of bilingualism and attention deficits (i.e., studies on either bilingualism or attention deficits, but not both); and (2) ineligible study design, including case studies and methodological or theoretical papers with no empirical data. Category 1 was also used to exclude studies on second language acquisition in a classroom context, as these studies do not consider the active use of two or more languages in everyday life, and thus do not align with our definition of bilingualism.

**Figure 1:**
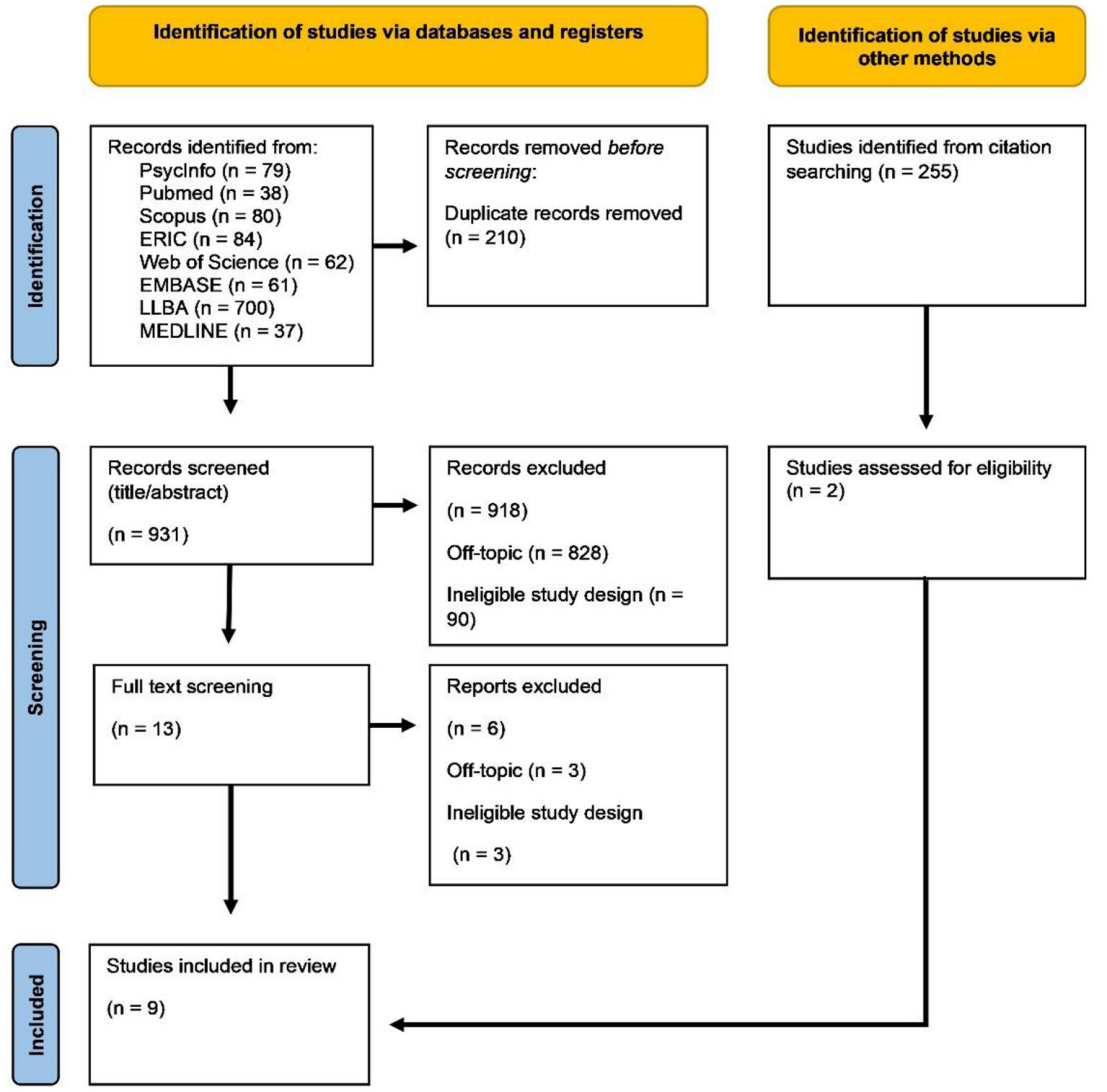
Combined results from first, second, and third search.

Details of the number of exclusions for each criterion can be found in the PRISMA flowchart (Page et al., 2021) in Figure 1.

After a full-text screening of the remaining studies, nine were deemed eligible. The three exclusions in this step were a conference abstract and two papers on classroom L2 acquisition. Two additional papers were found by looking through the Google citations of the eligible studies, increasing the total of included studies to 12. Of those 12 studies, 10 were published in peer-reviewed journals, while two were found in an unpublished Ph.D. dissertation which we acquired by contacting the author.

Additional searches were performed in October 2021 and May 2022, in order to locate potential new studies that had been published since the first search. 36 (October) and 116 (May) new studies were identified. All but one were excluded (off topic: 147, ineligible study design: 4). The new inclusion was a journal publication of one of the studies from the formerly unpublished PhD retrieved in the first search, thus replacing this study rather than adding to the list of inclusions.

After extracting key information from the 12 included studies and discussing their relevance with respect to their topic, methodology, and quality, three studies were excluded. Özerk et al. (2011) was excluded due to both its low number of participants and the fact that its main focus was on methodology and assessment. Ramos et al. (2019) was excluded, as while it did include bilingual children who were at high risk for ADHD, the focus of this study was to compare monolingual and bilingual subjects' usage of syntax and semantics rather than their executive function abilities or ADHD-related behavioural symptoms. Finally, Askari et al. (2019) intervention study was excluded because of methodological and statistical concerns as well as due to inconsistencies in the results section, where the presented data did not seem to match the conclusions presented. Therefore, the final number of included studies was nine.

## 3. Results

### 3.1. Study characteristics

#### 3.1.1. Participant information, variables, and analysis

Table 1 summarises general characteristics of the included studies. Across the nine studies included in the review a total of 2071 participants were tested. Of these, seven studies involved children (*N* = 1,823) ranging between 5 and 17 years of age with the exception of Goh et al. (2020), which tested younger children longitudinally from 2 to 4.5 years of age. Only two studies involved adults (*N* = 248); in both cases young adults typically in their twenties. All included studies were observational, examining the effects of independent variables on targeted variables. All studies included control variables, with age, gender, and socio-economic status (SES) being common, except for Mor et al. (2015), which did not include gender, and Hardy et al. (2021), which did not include SES. The way SES was operationalized varied between studies. Most studies used maternal education (Toppelberg et al., 2002; Bialystok et al., 2017; Sorge et al., 2017; Goh et al., 2020) or average number of parental years of schooling (Mor et al., 2015) as proxy for SES. Only two studies operationalized SES as a combination of affluence and parental education (Sharma, 2019; Sharma et al., 2022). The participating children in Chung-Fat-Yim et al. (2020)'s study were enrolled in a private school with high tuition fees, which they used as proxy for high SES. Six studies included verbal intelligence or language skills, and five studies included non-verbal intelligence. Several other control variables were measured, such as academic performance, ethnicity, and various immigration measures.

**Table 1:** Characteristics of the included studies.

**References**	**Age range (years)**	**Background/control variables**	**Independent variables**	**Outcome variables**	**Sample size**
Bialystok et al. (2017)	Average 21.8	Age; education; SES; verbal and non-verbal intelligence	Bilingualism (cat.) and ADHD diagnosis (cat.)	EF	168
Chung-Fat-Yim et al. (2020)	8–10	Age; SES; verbal intelligence (English and French) and non-verbal intelligence	Bilingualism (cont.) and ADHD symptoms (cont.)	EF	82
Goh et al. (2020)	Longitudinal (2, 3.5, 4.5)	Age; gender; SES; ethnicity; ODD diagnosis; cognitive ability	Bilingualism (cont.)	ADHD diagnosis at 4.5 years	408
Hardy et al. (2021)	6–17	Age; sex; ethnicity	Bilingualism (cat.) and ADHD symptoms (cont.)	EF and visual perception	511
Mor et al. (2015)	19–30	Age; SES; average number weekly hours video gaming	Bilingualism (cat.) and ADHD diagnosis (cat.)	EF	80
Sharma et al. (2022)	5–11	Age; gender; SES; structural language skill in English	Bilingualism (cat., cont.)	ADHD-related behaviour	394
Sharma (2019)	5–11	Age; gender; SES; verbal and non-verbal intelligence; structural language skill in English	Bilingualism (cat., cont.) and ADHD symptoms (cont.)	EF and ADHD-related behaviour	88
Sorge et al. (2017)	8–11	Age; education; SES; verbal and non-verbal intelligence	Bilingualism (cat., cont.) and ADHD symptoms (cont.)	EF	208
Toppelberg et al. (2002)	5–16	Age; gender; ethnicity, SES; immigration data	Bilingualism (cont.)	Attention difficulties	50

SES, socio-economic status; EF, executive function; cat., categorical; cont., continuous.

The included studies can be divided into two groups based on their outcome variables. Six studies measured cognitive abilities related to executive function (Mor et al., 2015; Bialystok et al., 2017; Sorge et al., 2017; Sharma, 2019; Chung-Fat-Yim et al., 2020; Hardy et al., 2021), while four studies looked at levels of attention problems (Toppelberg et al., 2002), ADHD-related behavioural symptoms (Sharma, 2019; Sharma et al., 2022) or the odds of receiving an ADHD diagnosis (Goh et al., 2020). The findings for these two groups of studies will be presented separately below.

The studies utilised various methods of statistical analyses. The most common analysis method was (stepwise) linear regression models, inputting control variables sequentially, with language status (categorical) or language ability (continuous) entered last. Separate regressions were run for each dependent variable, whether that be levels of ADHD symptoms/attention difficulties, or executive function measures (Sorge et al., 2017; Sharma, 2019; Chung-Fat-Yim et al., 2020; Hardy et al., 2021; Sharma et al., 2022). None of these studies reported the inclusion of random effects such as random intercepts or random slopes into the models. Other analysis methods included bivariate correlations (Toppelberg et al., 2002), ANOVAs (Mor et al., 2015; Bialystok et al., 2017) and moderated models (Goh et al., 2020).

#### 3.1.2. Bilingualism measures

The term “bilingual” is used across most studies, also to refer to participants who spoke or were exposed to more than two languages. Three studies (Mor et al., 2015; Bialystok et al., 2017; Hardy et al., 2021) analysed bilingualism as a categorical factor, i.e., participants were assigned to either a monolingual or a bilingual group. In Bialystok et al. (2017), participants were considered monolingual if they did not list a second language on the Language and Social Background Questionnaire (LSBQ; Luk and Bialystok, 2013) or reported only limited proficiency in another language. Participants were classified as bilingual if they reported a certain degree of proficiency and usage in another language. In Hardy et al. (2021), children were categorised as bilingual if parents answered that Spanish or Spanish and English were spoken in the home, monolingual if only English. Mor et al. (2015) used a Hebrew version (Prior and Beznos, 2009) of the Language Experience and Proficiency Questionnaire (LEAP-Q; Marian et al., 2007), which includes questions regarding language exposure and ratings of spoken language proficiency. Important to note is that the “monolinguals” in this study were highly proficient in both Hebrew and English, while the “bilinguals” spoke an additional third language.

Three studies (Sorge et al., 2017; Sharma, 2019; Sharma et al., 2022) analysed bilingualism as both categorical (monolingual vs. multilingual) and continuous factor (within their bilingual samples). Sharma (2019) and Sharma et al. (2022) used a language and family background questionnaire based on the Alberta Language Environment Questionnaire (ALEQ; Paradis, 2011). Children of caregivers who indicated their child as bilingual were categorised as bilingual. They were further asked to rate their children on speaking, understanding, reading and writing in all their languages. Information was also gathered on age of onset of bilingualism, and proportion of use of English vs. non-English in the home. A continuous composite language ability score was created from standardising these scores. Sorge et al. (2017) used the LSBQ (Luk and Bialystok, 2013) to quantify the children's language environment and usage.

Finally, three studies (Toppelberg et al., 2002; Chung-Fat-Yim et al., 2020; Goh et al., 2020) analysed bilingualism only as a continuous measure. Chung-Fat-Yim et al. (2020) used a later version of the LSBQ (Anderson et al., 2018). In Goh et al. (2020) a continuous measure of bilingualism was obtained by asking parents to give the aggregate proportion of input their baby received in all their languages, with all scores adding up to 100%. The proportion of time the less-heard languages were used was the measure of bilingualism (0 monolingual, 50—balanced bilingual). The sample in Toppelberg et al. (2002) comprised of English-Spanish speaking-children whose mothers, families and/or caregivers communicated solely or mainly in Spanish. Their language proficiency measure was based on parent ratings of children's use of English or Spanish in different settings and with different people.

#### 3.1.3. Attention/ADHD measures

The included studies differ in whether they analysed attention difficulties as a categorical or continuous factor. Three studies (Mor et al., 2015; Bialystok et al., 2017; Goh et al., 2020) used a categorical distinction between control participants and participants who had a previous ADHD diagnosis or showed clinical levels of attention deficits on diagnostic scales such as the Conners' Adult ADHD Rating Scales (CAARS-S:L; Conners et al., 1999) or the Diagnostic Interview Schedule for Young Children (DISC-YC; Fisher and Lucas, 2006). The remaining six studies used a continuous measure of attention difficulties. Three of these studies (Sorge et al., 2017; Sharma, 2019; Chung-Fat-Yim et al., 2020) used the Strengths and Weaknesses of Attention-Deficit/Hyperactivity Disorder Symptoms and Normal Behaviour Scale (SWAN; Swanson et al., 2012). Hardy et al. (2021) used the Swanson, Nolan and Pelham Parent & Teacher Rating Scale (SNAP-IV, Swanson, Nolan, and Pelham, Fourth Edition). Finally, two studies used diagnostic screeners. Sharma et al. (2022) used the ADHD subscale in the Social Skills Improvement System-Rating Scales (SSIS-RS; Gresham and Elliott, 2008). Toppelberg et al. (2002) used the Child Behaviour Checklist (CBCL) and Teacher's Report Form (TRF, Achenbach and Edelbrock, 1991), which includes an attention difficulties subscale.

### 3.2. Study results

This section summarises the results of the identified studies, starting with the studies targeting executive function skills or other cognitive abilities (see Table 2), and then turning to the studies examining the effect of bilingualism on ADHD-related behaviour and diagnosis (see Table 3).

**Table 2:** Effect of bilingualism and attention ability/ADHD status on executive function performance.

**Targeted EF**	**Task**	**References**	**Results**	**Bilingual advantage**
Interference control	Flanker task	Bialystok et al. (2017)	• RT: positive effect of bilingualism (cat.) and non-ADHD status (cat.) (when controlled for general processing speed), no interaction	+
		Chung-Fat-Yim et al. (2020)	• RT: No effect of bilingualism (cont.) and attention (cont.), no difference between bilingual and trilingual group	0
		Sorge et al. (2017)	Accuracy: • Complete sample: positive effect of attention (cont.), but not bilingualism (cat.), no interaction • Bilingual sample: positive effect of attention (cont.) and bilingualism (cont.), interaction (bigger boost of bilingualism for children with low attention) RT: no effect of attention (cont.) or bilingualism (cat./cont.)	+/0
	Inhibition-inhibition subtest (NEPSY-II)	Hardy et al. (2021)	• Negative effect of attention problems (cont.), no effect of bilingualism (cat.), no interaction	0
	Numeric Stroop	Mor et al. (2015)	• RT: No effect of language group (cat.) or attention group (cat.); three-way interaction between language group, attention group and congruency (greater interference effect for bilinguals with ADHD compared to control) • Accuracy: effect of attention group (cat.), but not language group (cat.)	0/(–)
	Simon Arrows	Mor et al. (2015)	• RT: No effect of language group (cat.) or attention group (cat.); three-way interaction between language group, attention group and congruency (greater interference effect for bilinguals with ADHD compared to control) • Accuracy: effect of attention group (cat.), but not language group (cat.)	0/(–)
	Colour-Word Stroop	Sharma (2019)	Complete sample: • RT: No effect of bilingualism (cat.) • Accuracy: marginal positive effect of bilingualism (cat.) on incongruent but not congruent trials Bilingual sample: • RT/accuracy: no effect of bilingual proficiency (cont.) or length of exposure to strongest non-English language	0/(+)
Response inhibition	Stop-Signal	Bialystok et al. (2017)	• SSRT: effect of attention (cat.) but not language group (cat.), interaction of attention and language group (for bilinguals, bigger difference between ADHD and control group; however, in ADHD group no effect of bilingualism)	0/**–**
		Sorge et al. (2017)	SSRT: • Complete sample: positive effect of attention (cont.) and bilingualism (cat.); no interaction; positive effect of cognitive ability • Bilingual sample: effect of attention (cont.), interaction between bilingualism (cont.) and attention (cont.) (bigger effect of bilingualism in children with strong attention abilities)	+
	Simon Arrows reverse block	Mor et al. (2015)	• RT: No effect of language group (cat.) or attention group (cat.) • Accuracy: effect of attention group (cat.), but not language group (cat.)	0
Working memory	Animal Sounds Monitoring Task	Sharma (2019)	• Complete sample: No effect of bilingualism (cat.) • Bilingual sample: no effect of bilingual proficiency (cont.) or length of exposure to strongest non-English language	0
	Frog Matrices Task	Sorge et al. (2017)	• Complete sample: positive effect of bilingualism (cat.), no effect of attention (cont.), no interaction, effect of age and cognitive ability • Bilingual sample: positive effect of bilingualism (cont.), no effect of attention, no interaction, effect of age, SES, and cognitive ability	+
Cognitive flexibility	Trail Making Task	Mor et al. (2015)	• RT/accuracy: no effect of language group (cat.) or attention group (cat.)	0
	Task Switching Paradigm	Mor et al. (2015)	Switching cost (difference in performance between switch and non-switch trials in mixed blocks): • RT: No effect of language group (cat.) or attention group (cat.) • Accuracy: effect of attention group (control more accurate than ADHD group); four-way interaction between attention group, language group, congruency (congruent-incongruent), and trial type (repeat-switch) (only monolingual controls have similar switch costs for congruent and incongruent trials) Mixing cost (difference in performance between single-task blocks and non-switch trials in mixed blocks): • RT: No effect of language group (cat.) or attention group (cat.) • Accuracy: effect of attention group (control more accurate than ADHD group)	0
	Global-Local Task	Sharma (2019)	Complete sample: • RT: no effect of bilingualism (cat.) • Accuracy: positive effect of bilingualism (cat.) Bilingual sample: • RT: no effect of bilingual proficiency (cont.) or length of exposure to non-English language • Accuracy: positive effect of bilingual proficiency, no effect of length of exposure to strongest non-English language	0/+
Decision making (reversal learning)	Child Iowa Gambling Task	Sharma (2019)	• Complete sample: no effect of bilingualism (cat.) • Bilingual sample: no effect of bilingual proficiency (cont.) or length of exposure to strongest non-English language	0
Delay tolerance	Delayed Reward Task	Sharma (2019)	• Complete sample: no effect of bilingualism (cat.) • Bilingual sample: no effect of bilingual proficiency (cont.) or length of exposure to strongest non-English language	0

EF, executive function; cat., categorical; cont., continuous; RT, reaction time. Bilingual advantage: “+”, positive effect; “–”, negative effect; “0”, no effect.

**Table 3:** Effect of bilingualism on ADHD-related behaviour/ADHD diagnosis.

**ADHD-related behaviour/diagnosis**	**References**	**Results**	**Bilingual advantage**
ADHD diagnosis at 4.5 y	Goh et al. (2020)	• Association between language delay and ADHD diagnosis only for primarily monolingual children • Children with no language delay: higher odds of ADHD diagnosis with increased bilingualism • Language-delayed children: no significant effect of bilingualism on ADHD diagnosis • No mediating effect of executive function (delay tolerance, cognitive flexibility)	–/0
ADHD-related behaviour	Sharma (2019, Study 1) and Sharma et al. (2022)	• Complete sample: small positive effect of bilingualism (cat.) when controlled for age, sex, and structural language skills • Bilingual sample: no effect of bilingual proficiency (cont.)	+/0
Inattentiveness, hyperactivity/impulsivity	Sharma (2019, Study 2)	• Complete sample: no effect of bilingualism (cat.) on inattentiveness or hyperactivity/impulsivity • Bilingual sample: no effect of bilingual proficiency (cont.) (oral or literacy proficiency in strongest non-English language, length of exposure to strongest non-English language) on inattentiveness or hyperactivity/impulsivity	0
Levels of attention difficulties	Toppelberg et al. (2002)	• Clinical subgroup: inverse correlation between bilingual proficiency (cont.) and attention problems	+

Cat., categorical; cont., continuous. Bilingual advantage: “+”, positive effect; “–”, negative effect; “0”, no effect.

An overview of the different executive function components and other cognitive abilities that have been assessed as outcome variables in the included studies can be found in Table 2. Most studies targeted interference control (*n* = 6), some looked at response inhibition (*n* = 3), working memory (*n* = 2), or cognitive flexibility (*n* = 2), while only one study tested decision making (*n* = 1) and delay tolerance (*n* = 1). In the following section we present the findings for each cognitive ability separately, assessing whether there is evidence for a bilingual advantage, disadvantage, or a null effect for that aspect of cognition, and whether bilingualism and attention levels interact. Almost all tasks avoided verbal elements in test trials, apart from the colour-Word Stroop task in Sharma (2019). The study does report, however, that any child unable to read the colour names prior to the task was not allowed to continue.

#### 3.2.1. Interference control

Interference control is the ability to ignore task-irrelevant, competing information. Half of the studies assessing interference control used a version of the Flanker task (Eriksen and Eriksen, 1974). In the critical part of the Flanker task, participants need to indicate the direction of a target stimulus when it is either surrounded by stimuli pointing in the same (congruent) or the opposite (incongruent) direction. Variables of interest are differences in accuracy and reaction times between congruent and incongruent trials.

In their study with four groups of young adults combining language group (monolingual/bilingual) and attention group (ADHD/non-ADHD), Bialystok et al. (2017) found that bilingual participants and participants with no ADHD diagnosis experienced a smaller interference effect once general processing speed was controlled for. Studying multilingual children, Chung-Fat-Yim et al. (2020) found no evidence that higher bilingual proficiency and better attention levels improved interference control. This null finding is in line with Sorge et al. (2017) who studied children of a similar age range and assessed bilingualism both as a categorical factor for the complete sample and continuous factor for the bilingual sample. However, with regards to accuracy in mixed blocks (congruent and incongruent trials mixed), Sorge et al. (2017) found that within the bilingual sample, a higher level of bilingualism improved accuracy and that this boost of bilingualism was more pronounced for children with low attention levels.

Mor et al. (2015) used two different tasks to measure interference control in young adults, a numeric Stroop (Hernández et al., 2010) and a Simon Arrows task (Bialystok et al., 2008). For both tasks, they did not find an overall effect of language group (monolingual/bilingual) or attention group (ADHD/non-ADHD) on reaction times. However, they report a significant three-way interaction between language group, attention group and congruency. Bilinguals with ADHD experienced greater interference from task-irrelevant information compared to bilinguals with no ADHD diagnosis. For the accuracy measure, only attention predicted performance, with ADHD participants being less accurate on incongruent trials than neurotypical controls.

Hardy et al. (2021) used the Inhibition-inhibition subtest from the NEPSY-II test battery (Korkman et al., 2007) to assess interference control in children with clinically significant levels of attention problems. Their results indicate that children with more attention problems experienced more interference from competing stimuli, but that bilingualism (monolingual/bilingual) did not have an effect. Sharma (2019) who used a Colour-Word Stroop (cf. Stroop, 1935) also did not find an effect of bilingualism (both measured categorical and continuous) on reaction times. However, this study reports a marginal effect of bilingualism on accuracy in incongruent trials, with bilingual children being slightly more accurate than monolingual ones.

To sum up, there is mixed evidence for a combined effect of bilingualism and attention levels on interference control, with some evidence for a bilingual advantage in children and adults (Bialystok et al., 2017; Sorge et al., 2017; Sharma, 2019), one study indicating a disadvantage for bilingual adults with ADHD (Mor et al., 2015), and a majority of null findings (Mor et al., 2015; Sorge et al., 2017; Sharma, 2019; Chung-Fat-Yim et al., 2020; Hardy et al., 2021).

#### 3.2.2. Response inhibition

Response inhibition is the ability to suppress a prepotent response. In the Stop-Signal task, used by Bialystok et al. (2017) and Sorge et al. (2017), participants are trained to quickly and accurately respond to a certain property of a stimulus (e.g., press “F” for a blue circle and “J” for a red circle on a keyboard). In the critical block, some stimuli are followed by an auditory “stop” signal at different intervals after stimulus onset, which requires participants to inhibit their response. The measurement of interest, the so-called Stop-Signal Reaction Time (SSRT), is calculated as the difference between the mean reaction time on “go” trials and the mean Stop-Signal delay, with a lower SSRT indicating better response inhibition.

Using the Stop-Signal task, Bialystok et al. (2017) found an effect of attention group (ADHD/non-ADHD), and a significant interaction between attention and language group. In particular, they found that for bilingual participants, people with an ADHD diagnosis had significantly longer SSRTs than their bilingual peers with no diagnosed attention difficulties. However, bilingualism did not turn out to be an additional burden for people with ADHD since the performance of monolingual and bilingual participants with ADHD did not differ. Using a similar task with children, Sorge et al. (2017) found that both higher attention levels and being bilingual improved response inhibition, with no interaction between these two factors. Within the sample of bilingual children, better attention levels again predicted better performance. In addition, a significant interaction with level of bilingualism indicated that children with strong attention abilities benefitted more from bilingual experience and proficiency than children with weaker attention abilities. Mor et al. (2015) tested habitual response inhibition using a reverse Simon Arrows task, in which participants needed to press the response button in the direction opposite to the one indicated by the arrow. They did not find an effect of attention group or language group on reaction times, and only an effect of attention group on accuracy, indicating that participants with ADHD tended to make more mistakes.

In sum, the available evidence suggests that response inhibition in adults is affected by attention levels, but not bilingualism (Mor et al., 2015; Bialystok et al., 2017). For children, one study indicates that being bilingual might positively affect response inhibition, especially when children have strong attention levels (Sorge et al., 2017).

#### 3.2.3. Working memory

The combined effects of attention and bilingualism on working memory capacity have so far only been assessed in children, with again mixed results. Sharma (2019) created an Animal Sounds Monitoring Task [based on Miyake et al. (2000)'s Tone Monitoring Task], which required children to monitor different animal sounds and to press a designated button when the sound of each particular animal was presented for the third time. No difference in auditory working memory capacity was observed between monolingual and bilingual children, neither effect of bilingual proficiency nor length of exposure within the bilingual sample.

In contrast to that, Sorge et al. (2017) found a bilingual advantage in spatial working memory capacity for both the complete sample and the bilingual sample. They used a Frog Matrices Task (Morales et al., 2013), where children needed to recall how a frog jumped between ponds arranged in a 3 × 3 grid. Bilingual children outperformed their monolingual peers in this task, and within the group of bilingual children a higher degree of bilingualism was related to better working memory capacity. Attention ability did not affect outcomes.

The divergence in findings between Sharma (2019) and Sorge et al. (2017) could be due to differences in their samples or the fact that different aspects of working memory (auditory vs. spatial working memory) were assessed, that could be differentially influenced by bilingualism.

#### 3.2.4. Cognitive flexibility/Shifting

Cognitive flexibility refers to the ability to shift between different concepts or task rules and to adapt the corresponding behavioural response accordingly. Mor et al. (2015) used two tasks to assess cognitive flexibility in adults. In a Hebrew version of the Trail Making Task (Reitan and Davison, 1974), participants were asked to connect numbers and Hebrew letters in alternating order (e.g., 1-“Alef,” 2-“Bet,”). In a Task Switching Paradigm, participants needed to switch between sorting figures according to their shape and their colour, depending on a task cue presented visually before each trial. While Mor et al. (2015) did not find an effect of language or attention group on performance on the Trail Making Task, they report several significant results for the Task Switching Paradigm. Looking at switching costs, i.e., the differences in performance when people had to switch between the shape and colour task compared to when no switching was required, they detected that people with ADHD tended to make more mistakes than the control group. In addition, they report a four-way interaction between attention group, language group, congruency (same vs. different response required for colour and shape task), and trial type (repeat vs. switch), in the sense that only participants in the monolingual control group had similar switch costs for congruent and incongruent trials. For mixing costs, defined as the difference in performance between single-task blocks and non-switch trials in mixed blocks, they also found people with ADHD to be less accurate than controls.

Sharma (2019) measured cognitive flexibility with a Global-Local Task (cf. Navon, 1977) in which children needed to shift between paying attention to the overall global shape of a figure and the local shapes it consists of. Sharma (2019) reports an effect of bilingualism on accuracy for both the complete and the bilingual sample. Bilingual children were more accurate than their monolingual peers, and a higher bilingual proficiency was connected to better task performance. However, since attention levels were not included in the model, it is unclear whether both attention and bilingualism are independent predictors of cognitive flexibility.

Taken together, the evidence on how attention abilities and bilingualism affect cognitive flexibility is still sparse and requires further investigation.

#### 3.2.5. Decision-making and delay tolerance

Decision-making and delay tolerance have been assessed in only one of the included studies as outcome variables (Sharma, 2019, but see also Goh et al., 2020 where delay tolerance is used as moderating variable). Decision-making skills were tested *via* a child version of the Iowa Gambling Task (Garon et al., 2006), that required children to decide from which of four decks a card should be turned over, with some decks containing more “good” cards than others. Delay tolerance, the ability to wait for a higher reward, was assessed with a Delayed Reward Task (Cherek et al., 1997). Performance on neither task was related to bilingualism, either for the complete sample or the bilingual subsample.

#### 3.2.6. ADHD-related behaviour

After reviewing the effects of bilingualism on cognitive performance, we now turn to studies looking at ADHD-related symptoms, as reported by parents and teachers (see Table 3). Sharma et al. (2022) assessed ADHD-related behaviour with the ADHD subscale of the SSIS-RS parent form (Gresham and Elliott, 2008), which gives a composite score for several ADHD-related symptoms of inattentiveness, hyperactivity/impulsivity, and oppositional defiant behaviour. They report a small significant effect for bilingualism as a category on levels of ADHD-related behaviour, such that bilingual children showed slightly less ADHD-related behaviour than their monolingual peers, when age, sex, SES, and structural language skills were controlled for. However, within the group of bilingual children, a higher level of bilingual ability (composite of oral proficiency, literacy proficiency, and bilingual use with caregivers) did not predict less ADHD-related behaviour. Sharma (2019, study 2) tested a sample of children both monolingual and bilingual, who scored ≤ −1 SD and ≥ +1 SD on the SSIS-RS ADHD subscale (as reported in Sharma et al., 2022), using the SWAN (Swanson et al., 2012) to assess ADHD symptomatic behaviour. No relation was found between bilingualism either as a category or continuous measure on inattentiveness or hyperactivity/impulsivity symptoms.

Focusing on bilingual Spanish-English-speaking children referred to psychiatric services, Toppelberg et al. (2002) looked among others at the relationship between bilingual language proficiency and attention problems. For children in the clinical range (CBCL score above the clinical cut-off), limited bilingual skills were associated with heightened attention problems, also when controlling for IQ. For the complete sample, the negative correlation between bilingualism and attention problems was still present, but weaker.

In sum, the limited evidence indicates that exposure to multiple languages could have a positive effect on ADHD-related behaviour such as inattentiveness or hyperactivity/impulsivity symptoms.

#### 3.2.7 ADHD diagnosis

Goh et al. (2020)'s study stands out in its design and methodology from the other identified studies, as they used a longitudinal design examining the prospective association of language delay at 2 years to ADHD diagnosis at 4.5 years. They found that for children primarily exposed to a single language, language delay was significantly associated with increased odds of getting an ADHD diagnosis. For children who did not show signs of language delay at 2 years, higher bilingual exposure increased the odds of getting an ADHD diagnosis at 4.5 years. By contrast, for language-delayed children, increased bilingual exposure did not moderate the association of language delay to ADHD, with a tendency towards increased bilingual exposure reducing the odds of an ADHD diagnosis later in childhood. Executive function skills, as measured by a delay tolerance and a cognitive flexibility task, did not mediate the link between language delay in interaction with bilingualism on ADHD diagnosis.

## 4. Discussion

This review identified and systematically summarised the available scientific evidence on how bilingualism affects cognitive abilities and ADHD-related behaviours/symptoms in adults and children with high and low attention levels. With only nine identified studies in the literature, the topic is to date not well-studied, and the total number of participants is limited. In addition, there is a big variability in the included studies concerning design and methodology. Not only do the identified studies assess different types of executive functions (e.g., working memory, interference control, cognitive flexibility), the tasks to measure them and the reported outcome variables also differ across studies. Furthermore, there are considerable differences in the conceptualisation and operationalisation of bilingualism and attention levels as continuum, category, or both. This variability makes it difficult to compare and synthesise evidence across studies.

Bilingualism and attention are dimensional constructs, with both having multiple underlying contributing factors, as is evident in the instruments used to assess them. Similarly, clinical ADHD constitutes the end of a dimension or dimensions, that falls along a continuum with the behaviour of neurotypical individuals. It follows that both bilingualism and attention abilities/difficulties, are better understood and explored as dimensional rather than categorical (Coghill and Sonuga-Barke, 2012; Luk and Bialystok, 2013; Roberts et al., 2015). Categorical approaches are no doubt important and useful, for instance for deciding who should be prioritised for intervention or for answering the question whether there is something qualitatively different about being bilingual that influences attention. On the other hand, a dimensional approach may reveal for instance for bilingualism what specific components (e.g., oral or literacy proficiency, age of onset, frequency of use, domains of use), if any, influence levels of behaviour across the different domains of ADHD.

Examining the overall evidence, no clear pattern emerged that individuals with attention deficits show systematic bilingual advantages or challenges in specific executive functions (interference control, response inhibition, working memory, cognitive flexibility), related cognitive abilities (decision-making, delay tolerance), or ADHD-related behaviour. However, among many null findings, several studies reported significant effects of bilingualism, sometimes in interaction with attention levels or ADHD status. For instance, Mor et al. (2015)'s study suggests that for people with ADHD being bilingual might be an extra burden, negatively affecting interference control. However, for this study, it should be borne in mind that the study samples involved might be more accurately described as bilingual vs. trilingual as previously mentioned in the section on bilingual measures.

Two studies found some initial evidence that bilingualism could lead to improvements in ADHD-related symptoms (Toppelberg et al., 2002; Sharma et al., 2022). However, to date there is no theory linking bilingual language experience to behavioural difficulties in conversation such as turn-taking and interrupting. Sharma et al. (2022) suggest that bilingual children's improved perspective-taking skills, as reported in Fan et al. (2015), could play a role. More research is needed to explore potential links between bilingualism and behavioural aspects related to inattention, hyperactivity, and impulsivity.

Some significant effects reported in the identified studies might have been due to a confound between bilingualism and SES. Previous research has shown that lower SES is associated with ADHD (Russell et al., 2015; Michaëlsson et al., 2022). In two of the studies examined, SES may have favoured results for bilinguals over monolinguals. In Bialystok et al. (2017), where bilinguals with ADHD scored better on hyperactivity/impulsivity scales than their monolingual counterparts, their SES was also higher than those of monolinguals with ADHD. In Sharma et al. (2022), bilingual children showed significantly lower levels of ADHD-related behaviour, but they had also a slightly higher SES than monolingual children. In addition, the way SES was operationalized varied considerably between studies, from using a single indicator such as maternal education to using an aggregate of affluence and parental education. We recommend that future studies should carefully consider how to operationalize SES, what components to measure (e.g., income, poverty, wealth, parental education, parental occupation), and whether to include them in the analysis separately or combined (cf. Ensminger and Fothergill, 2003; Duncan et al., 2015). Also other background variables such as gender, age, education, IQ, and structural language abilities need to be measured and compared between groups to prevent any confounds. While most studies assessed and reported at least some of these background factors, they were typically not included as covariates in the statistical analysis.

In light of the overall extremely limited number of available studies, any reported positive or negative effects of bilingualism in people with attention deficits needs to be seen as preliminary and awaits replication. In addition, there are several limitations of the included studies. It remains unclear whether the studies were strictly confirmatory or included explorations of the data, with multiple testing inflating the type I error rate considerably (Ioannidis, 2005). Furthermore, the chance of false-positive outcomes is increased by the statistical approach most studies have selected, analysing their data with correlations, ANOVAs or linear models without a maximal random effects structure (cf. Barr et al., 2013). It therefore needs to be seen whether the significant findings reported in some of the papers can be reproduced with a more suitable type of statistical analysis, taking complex dependencies between observations into account.

Taken together, we did not find support for the hypotheses that bilingualism has systematic positive or negative cognitive or behavioural effects on people with attention deficits, which makes the null hypothesis to date the most plausible candidate. The fact that no clear pattern across the included studies emerged suggests that significant effects might be due to characteristics of individual study samples, or the type of analysis selected rather than being generalizable effects. However, since the current evidence is limited and variability between studies is high, further research on this topic is needed, preferably with pre-registered design and analysis plans. It is particularly important to carefully measure different aspects of bilingualism (e.g., language proficiency, language use in different domains) and attention abilities (e.g., ADHD diagnosis, ADHD-related symptoms) to better understand the conditions under which potential differences in executive function performance emerge (cf. Pot et al., 2018; Grundy, 2020).

Based on the current evidence, exposure to more than one language does not seem to impair the cognitive functioning of people with ADHD or intensify inattention symptoms. This means that people with ADHD do not experience an additional cognitive burden or an added disadvantage by acquiring and using multiple languages. These findings are important beyond the scientific community. There is a widespread fear that children who already face developmental challenges might be overburdened by the demands of learning one or more additional languages. It is not uncommon that parents of children with neurodevelopmental disorders get professional advice that it might be harmful for their child's development if they are exposed to more than one language (e.g., Kay-Raining Bird et al., 2012). In line with Uljarević et al. (2016), we would like to point out that such general recommendations are not backed up by the current state of research. This notwithstanding, there is still a lot we do not know about the bilingual language development of children with developmental disorders [see Kay-Raining Bird et al. (2016) for a review]. Further research is needed to better understand the challenges that children with attention deficits face when acquiring multiple languages, especially because language and communication disorders are common in this group (Green et al., 2014; Tannock, 2018).

On the other hand, bilingualism also does not “train” attention abilities of people with attention deficits. The results of this review add to the scientific debate on a bilingual advantage by presenting evidence from a subgroup of the population that has attention deficits and might therefore be particularly receptive for attention-related effects of bilingualism. The evidence for the null hypothesis in this population aligns with recent meta-analyses on neurotypical adults and children that did not provide support for domain-general cognitive benefits of bilingual speakers (Lehtonen et al., 2018; Lowe et al., 2021).

Potential bilingual benefits are therefore unlikely to be responsible for lower rates of ADHD diagnosis and treatment in children from minority-language backgrounds (e.g., Slobodin and Masalha, 2020). There may be several other explanations, such as cultural differences in caregiver expectation of development, for instance, not being familiar with ADHD, and also limited knowledge how to access assessment or treatment (Stevens et al., 2004; Rothe, 2005; Eiraldi et al., 2006). Mor et al. (2015) mentioned these as possible explanations for the make-up of their bilingual sample. That is, that only the most severe cases of ADHD among bilinguals appeared to be accounted for, as those with less severe difficulties were unaware of ADHD or decided against seeking help to avoid additional stigma.

In line with previous research on ADHD (Willcutt et al., 2005), the included studies show that children and adults with attention deficits can perform significantly below neurotypical controls in executive functions including interference control, response inhibition, and cognitive flexibility. Interestingly, the effect of attention level was most visible in the accuracy measures. The general tendency across the included studies was that people with limited attentional resources were equally fast—or sometimes slower—than controls, but less accurate. Impairments in the optimisation of the speed-accuracy trade-off have been previously linked to ADHD (Mulder et al., 2010).

## 5. Conclusion

There is to date little evidence that bilingualism affects cognition or behavioural symptom presentation in children and adults with attention deficits. If bilingual effects are real, their effect sizes will likely be small and practical implications for affected individuals will be limited. Especially, it is unlikely that they will outweigh the clear social and linguistic advantages of bilingualism. Given the impact on individuals, families, and society, however, it is important to continue to investigate why differences exist in assessment, diagnosis, and treatment of ADHD in minority communities, and formulate strategies to address these.

## Data availability statement

The original contributions presented in the study are included in the article/supplementary material, further inquiries can be directed to the corresponding author.

## Author contributions

FK, SC, and MG contributed to conception and design of the study. SC performed the search in relevant databases. SC and FK screened the papers. FK and CS wrote the first draft of the manuscript. All authors commented on the draft and approved the submitted version.
